# Research Progress of Thermosensitive Hydrogel in Tumor Therapeutic

**DOI:** 10.1186/s11671-021-03502-5

**Published:** 2021-03-04

**Authors:** Nian Ma, Zhihui Yan

**Affiliations:** 1 The People’s Hospital of Danyang, Affiliated Danyang Hospital of Nantong University, Danyang, 212300 Jiangsu Province China; 2 The Affiliated Huai’an Hospital of Xuzhou Medical University and The Second People’s Hospital of Huai’an, No.62, Huaihai Road (S.), Huai’an, 223002 China

**Keywords:** Thermosensitive, Hydrogel, Tumor, Thermotherapy

## Abstract

Compared with traditional tumor therapy strategies, hydrogel as a drug reservoir system can realize on-demand drug release and deep tissue penetration ability. It also exhibits great tumor-site retention to enhance the permeability and retention effect of tumor treatment. This can significantly overcome the drug's resistance and severe side effects. Inorganic/organic composite hydrogel has attracted wide attention due to its combined effects, enhancing therapeutic effects against various kinds of tumors. In situ injectable hydrogel can securely restrict the drugs in the lesion sites without leakage and guarantee better biosafety. Moreover, hydrogel possesses interconnected macropores which can provide enough space for nutrient transport, cellular activity, and cell–cell interactions. Thermal therapy is an effective strategy for tumor therapy due to its minimal invasiveness and high selectivity. Because the location temperature can be precisely controlled and helps avoid the risks of destroying the body's immune system and ablate normal cells, thermal therapy exhibits significant treatment outcomes. Nonetheless, when the cellular temperature reaches approximately 43 °C, it causes long-term cell inactivation. Based on these merits, thermosensitive hydrogel formulation with adaptive functions shows excellent efficacy, unlimited tissue penetration capacity, and few deleterious side effects. Furthermore, the thermosensitive hydrogel has unique physical properties under the external stimuli, which is the ideal drug delivery system for on-demand release in tumor treatment. This article will review the state of the thermosensitive hydrogel in clinic application for cancer therapy.

## Introduction

Over the past years, researchers have focused their attention on 3D biomaterials since the cross-linked macropores provide enough space for nutrient transport, cell activity, and cell–cell interactions [1]. As the ideal drug carriers, the hydrogel has minimal invasiveness. It can form desired shapes to meet the requirement of irregular lesion sites in cancer therapy [2]. The traditional hydrogel is usually fabricated through physical interaction or chemical binding of constituting polymer, which has minimal effects on their function [3]. Hydrogel as a drug delivery system should respond to endogenous/exogenous stimuli, thereby ensuring the drug on-demand release in the lesion sites and reducing unnecessary side effects on normal tissues [4]. Functional inorganic nanomaterial incorporated into the hydrogel can significantly overcome the intrinsic limits, which has other fascinating properties and remarkably improves stimuli-responsive therapeutic efficacy [5–7].

Thermal therapy has the advantages of local temperature controlling and minimal invasiveness, which became a novel therapy approach after chemotherapy, radiotherapy, and surgical intervention in current tumor treatment [8]. Based on hydrogel inorganic material mediated thermotherapy with the unique physical feature under certain stimuli, it is the ideal agent delivery platform for on-demand drug dose therapy in lesion sites [9–11]. Compared with traditional synergistic therapeutic approaches (chemo/radiotherapy, chemo/photodynamic therapy, and photodynamic/photothermal therapy), thermosensitive hydrogel loaded with antitumor drugs. This can help penetrate drugs into deep tissues, form desired shapes to fill the irregular tissues, and promote wounds healing [12]. Also, mild temperature heating can enhance chemotherapy outcomes by improving cytomembrane's permeability to increase cellular uptake of drugs and control drug release from the hydrogel. When the cellular temperature exceeds 41 °C, protein denaturation and temporary cell inactivation occur, and this lasts for several hours. When the temperature reaches approximately 43 °C, it causes long-term cell inactivation [13]. Moreover, injecting thermosensitive hydrogel in situ into the lesion sites can avoid the risk of drug accumulation at the liver and spleen to improve therapy outcomes and guarantee better biosafety in vivo [14].

The benefit of the thermosensitive hydrogel in the clinic can facilitate administration, improve therapeutic efficacy in the lesion region, and reduce unnecessary damage to normal tissues, thereby improving patient compliance. This article will summarize some thermosensitive hydrogels to improve disease treatment and make the current state of the hydrogel in clinic application.

### Magnetic Hyperthermia Hydrogel

It is well known that the doping concentration of inorganic nanoparticles into the hydrogel can inevitably affect the intrinsic hydrogel properties, which usually shows dose-dependence [15]. The high concentration of agents would enhance the therapeutic efficacy. However, unnecessarily it deteriorates the rheological properties of the hydrogel, resulting in burst release, uncontrolled treatment, and severe side effects on normal tissues [16].

**Fig. 1:**
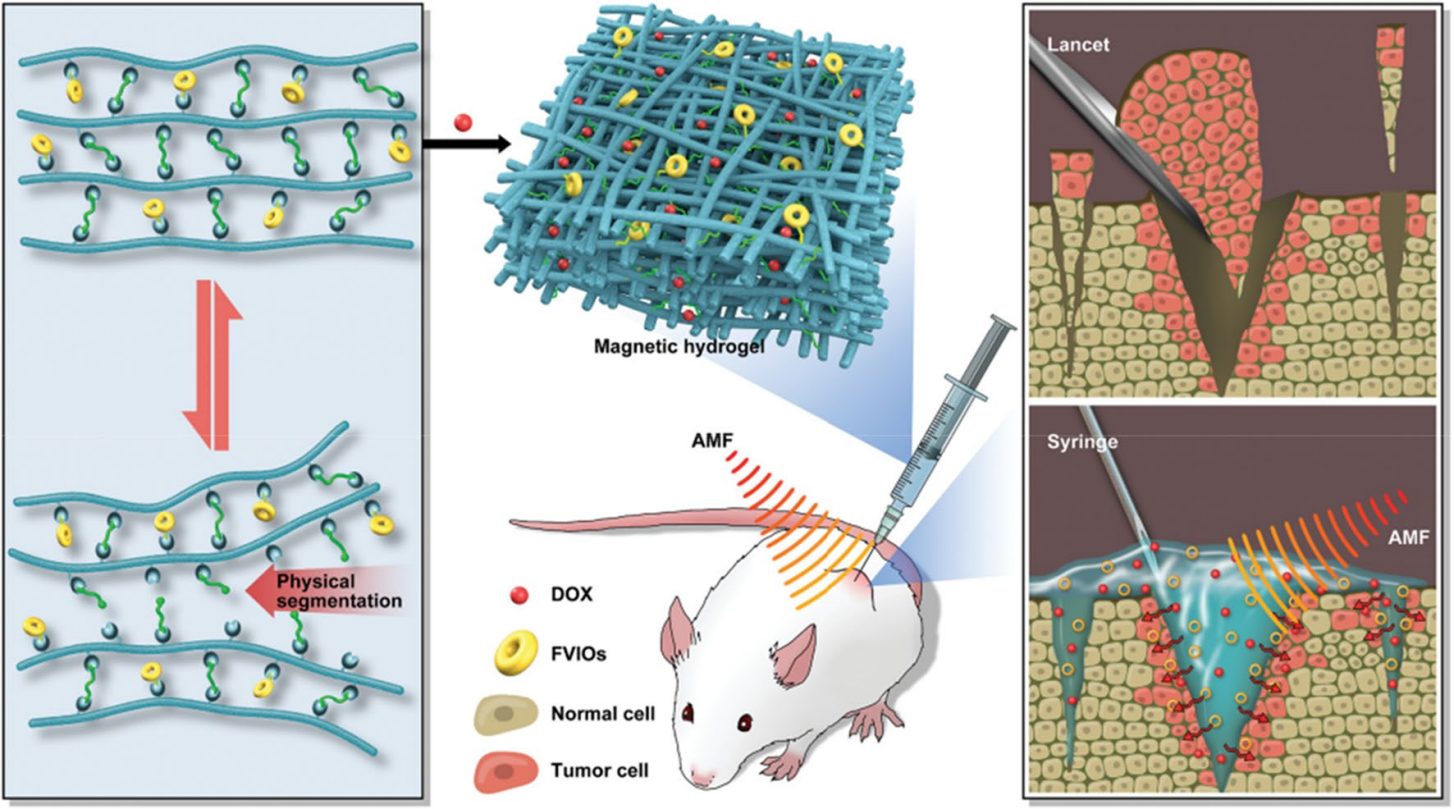
Illustrates FVIO-functionalized magnetic hydrogel with optimal adaptive functions for breast cancer postoperative recurrence prevention [18]. Copyright 2019 Adv. Healthcare Mater

It is challenging to fabricate high-performance nanoparticle hydrogel, which should balance hydrogel's intrinsic properties and associate the functions associated with the inorganic nanoparticle loading process. This contradiction is very obvious in designing magnetic hydrogel in the synergistic thermos-chemotherapy for highly efficient postsurgical treatment [17]. This shortcoming would be effectively overcome, providing good rheological properties and sufficient heating efficiency. This is based on glycol-chitosan, difunctional telechelic poly (ethylene glycol) (DT-PEG), and ferromagnetic vortex-domain iron oxide (FVIOs) as the raw materials (Fig. 1) [18]. Compared with traditional magnetic hydrogel, the obtained magnetic hydrogel overcomes the side effects and exhibits remarkable rheological properties and high heating convert ability in an alternating magnetic field [19]. Further, this self-adapting magnetic hydrogel regulates the drug in a long-term sustainable manner. It directly targets the lesion sites. Magnetic hyperthermia can promote the internalization of a drug, eventually causes cancer cells apoptosis and reducing tumor size. The FVIO-incorporated hydrogel has the features of self-healing, fast gelation, and self-confirming ability, which can satisfy synergistic thermos-chemotherapy and provide an alternative strategy for addressing the unmet clinical need. This work underlines the potential promise for the precision of the injection sites. It enhances the magnetic hyperthermia efficiency for xenograft tumor treatment.

### Near-Infrared Light Absorption Hydrogel

Photothermal therapy (PTT) has attracted wide attention due to its unbeatable advantages in cancer treatment, including control treatment and satisfactory cancer eradication outcomes [20–22]. However, conventional PTT has poor penetration into the site of the deep lesions, bring harmful effects on therapy. Chemotherapy and PTT synergistic strategy might be a well-pleasing candidate to enhance the tumor therapeutic efficacy [23].

Various photothermal materials have been widely exploited as drug delivery carriers or coupling reagents for cancer therapy, including metal–organic frameworks and carbon dots [24–27]. Among these materials, conjugated polymer dots (Pdots) are biocompatible, degradable, and nontoxic biomaterial with easy functionalization. These are small in size and extraordinary photophysical properties [28–31]. More importantly, Pdots with strong optical absorption properties and photostabilities in the near-infrared (NIR) light window are satisfying agents for PTT and photoacoustic imaging (PAI) [32–34]. Iohexol is an efficient and safe contrast agent approved by U.S. Food and Drug Administration for body computed tomography (CT) imaging [35]. However, the time of Iohexol for CT imaging is very short, and this inevitable shortcoming limits Iohexol widely used in the clinic [26]. Grating iohexol into Pdots-DOX-based thermosensitive hydrogel can successfully overcome this disadvantage of iohexol for enhanced CT imaging ability. This makes hydrogel an excellent candidate used in cancer theranostics.

**Fig. 2:**
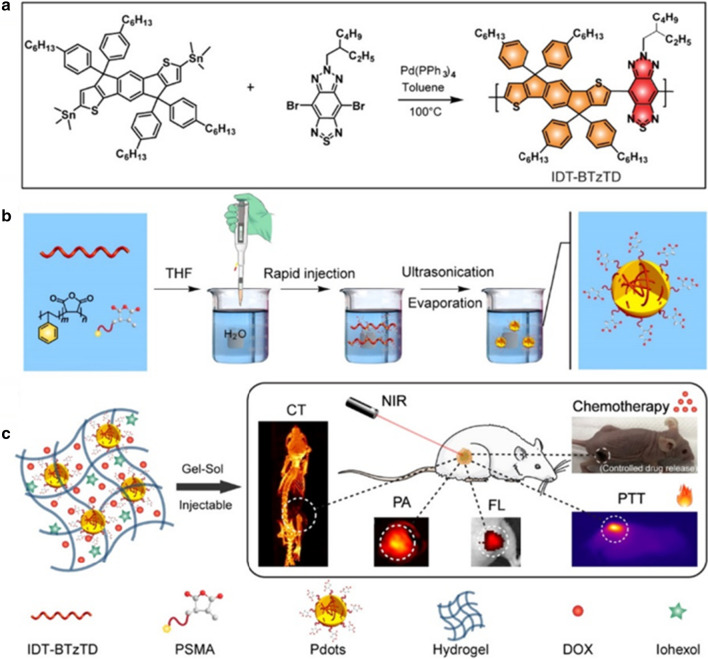
**a** Schematic of the fabrication of conjugated polymer IDT − BTzTD. **b** Schematic of the fabrication of IDT − BTzTD Pdots. **b** Schematic of the Pdots − DOX − iohexol@hydrogel for trimodal FL/PA/CT imaging-guided synergistic chemo-photothermal cancer therapy [36]. Copyright 2020 ACS Appl. Mater. Interfaces

Based on these merits, Men et al. introduced a multifunction Pdots@hydrogel drug delivery platform with good biodegradability, strong NIR absorption ability, high photothermal conversion efficiency, and controlling drug release, well-pleased CT/PA/fluorescence imaging ability, and enhanced tumor therapeutic outcomes (Fig. 2) [36]. The obtained NIR light-mediated Pdots-DOX-iohexol@hydrogel system exhibits strong photothermal effects. It achieved dose-control chemotherapy by interval NIR light irradiation, superior tissue penetration, and minimal invasion in cancer treatment, thus inhibiting tumor growth. More importantly, the nanoengineering modality for the Pdots-DOX-iohexol@hydrogel possesses excellent CT/FL/PA imaging ability and high biocompatibility for cancer detection. Therefore, the concept of integrating various diagnostic/therapeutic agents into one system can be potentially applied to various perspectives of disease therapy in the clinic.

### Photothermal Effects Bifunctional Hydrogel

At present, the treatment of bone tumors mainly depends on surgical intervention and chemo/radiotherapy synergetic approaches, which significantly improves patients' survival rate [37]. However, surgical intervention always causes bone defects. It incompletely removes tumor cells, making bone tissues hard to heal by themselves, and residual cancer cells proliferate within several days. Therefore, it is significant to develop a biomaterial with tumor therapy and simultaneously promote bone regeneration after surgery.

**Fig. 3:**
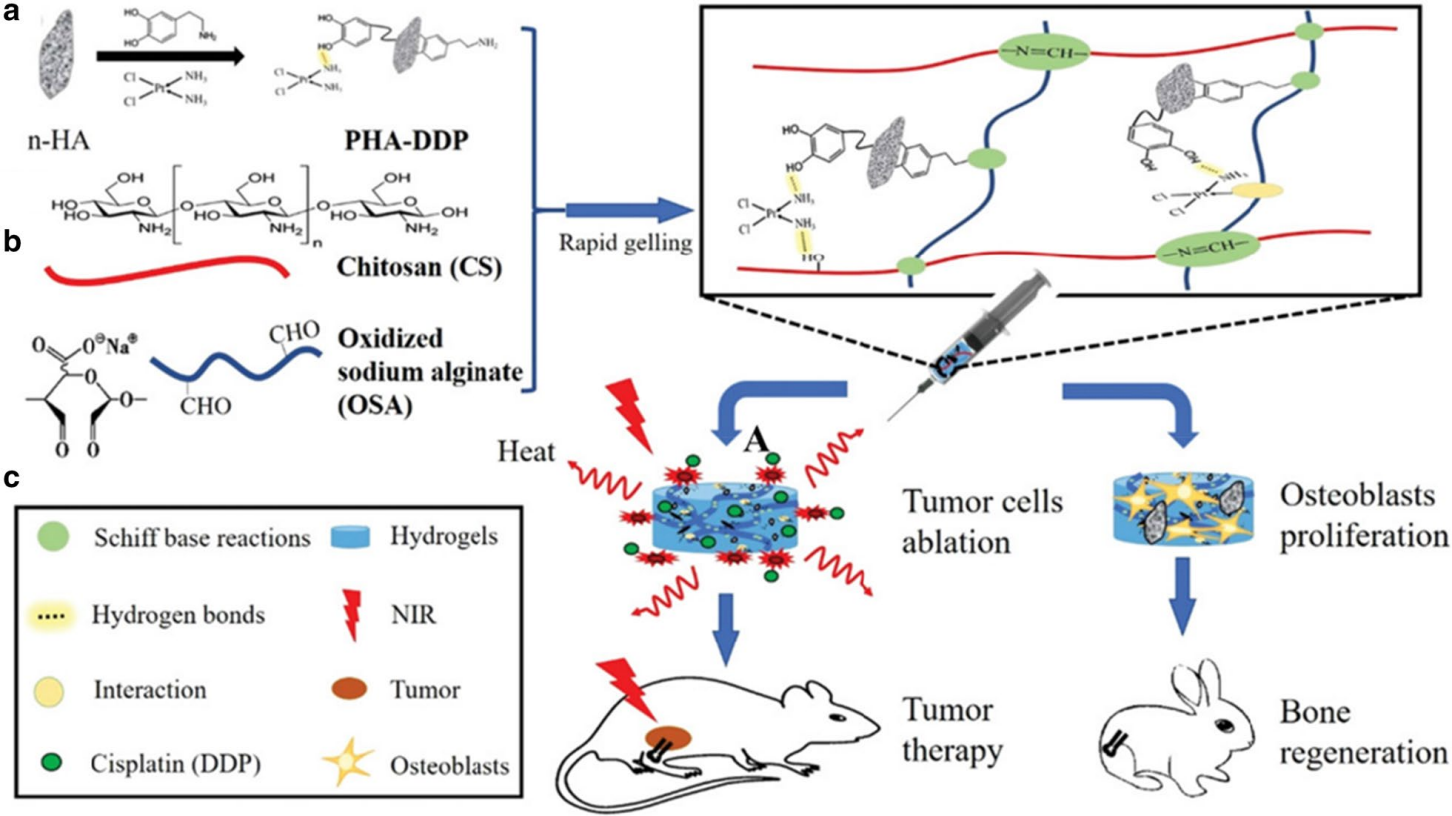
Schematic illustration of the formation of bifunctional OSA-CS-PHA-DDP hydrogels and bioapplication [40]. Copyright 2019 Macromol. Biosci

Injectable hydrogel as a promising alternative approach can form desired shapes to fill defects tissues. Its components are very similar to bone tissues for improving osteogenic ability [38]. The injectable hydrogel applied in bone tissue engineering should be slow enough to meet surgical handing and simultaneously be fast enough to realize stability and function after injection in vivo [39]. In order to solve these issues, Luo and his co-works provided a novel bifunctional injectable hydrogel. This hydrogel used polydopamine (PDA) to modify nano-hydroxyapatite (n-HA) and immobilize cisplatin (DDP) to fabricate PHA-DDP particles. It was then introduced PHA-DDP particles into the Schiff based on the reaction system between chitosan (CS) and oxidized sodium alginate (OSA) (Fig. 3) [40]. Nano-hydroxyapatite (n-HA) played an important role in bone formation, which is the major inorganic material in bone tissues and composed of calcium and phosphorus elements [41]. Mussel-inspired PDA as the ideal candidate for photothermal agents has good biocompatibility and biodegradability and has abundant functional groups. Mussel-inspired PDA easily deposits on various substances, such as loading antitumor drugs (cisplatin, DDP) through hydrogen bonding or other interactions [42–44]. Additionally, n-HA was modified into PDA to obtain PDA decorated n-HA (PHA), improving cell adhesion and proliferation [45].

The successfully obtained OSA-CS-PHA-DDP injectable hydrogel has excellent PDA photothermal effects of inhibiting tumor growth via local hyperthermia under laser irradiation. Further, mild photothermal effects can improve the permeability of cytomembrance to increase the cellular uptake of antitumor drugs. They can destroy hydrogen bond interactions between DDP and PDA to improve drug release and enhance tumor treatment effects. More importantly, PDA's abundant functional group can promote bone mesenchymal stem cell proliferation and adhesion and further facilitate new bone tissue formation. This bifunctional hydrogel integrates tumor treatment with bone regeneration based on these properties. It shows a promising approach for tumor-related bone defects in the clinic.

### PTT/PDT-Responsive Agarose Hydrogel

Tumor vascularity has poor integrity of structure, resulting in insufficient oxygen supply in tumor regions. Hypoxia condition causing an acidic tumor microenvironment by increasing production of lactic acid via anaerobic glycolysis [46]. Thus, hypoxia and low pH are the common features of tumor microenvironment severely compromising therapeutic efficacy.

Photothermal therapy destructs tumor tissues based on local hyperthermia mediated by photothermal agents under laser irradiation [47]. Thus, various kinds of photothermal agents have been developed to satisfy PTT performance [48]. However, most of them still have some drawbacks in clinical application, such as non-degradability, low biosafety and complex synthesis progress. Humic acid (HA) has excellent photothermal conversion ability and photoacoustic (PA) imaging, which is extracted from biochemical humification of animal and plants matter and it has attracted increasing attention in PTT [49]. Meanwhile, photodynamic therapy (PDT) is another effective strategy for tumor therapy by utilizing the oxygen reactive species (ROS) generated from oxygen molecules in the presence of photosensitizers (PS) under laser excitation [50]. Chlorin e6 has high ROS production yield and low dark toxicity, which has been widely used in PDT [51]. But, intrinsic hypoxia microenvironment can compromise therapeutic effects during PDT progress.

**Fig. 4:**
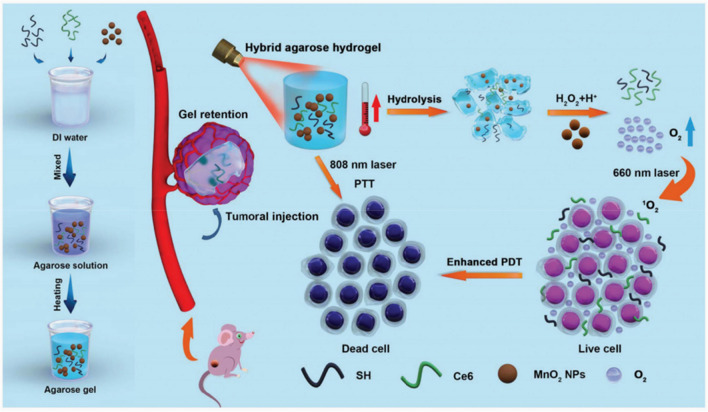
Schematic diagram of the synthesis process and working principle of the agarose@SH/MnO_2_/Ce6 hydrogel. Effective tumor inhibition was accomplished through enhanced photo-induced tumor therapy on the basis of the relief of tumor hypoxia [53]. Copyright 2020 Biomater Sci

LMP agarose hydrogel melts at the temperature above 65 °C and sol-to-gel transition begins at the temperature under 25 °C during the cooling process, which displays great potential for on-demand drug administration by precisely regulating various temperature [7, 52]. Therefore, rational designed and functionalized LMP agarose hydrogel is a promising approach to realizing high drug bioavailability and enhancing therapeutic outcome through one single injection. As Fig. 4 is shown, Hou et al. provided a novel “co-trapped” approach by simultaneously incorporating SH, Ce6 and MnO_2_ nanoparticles into low melting point (LMP) agarose, and the obtained agarose@SH/MnO_2_/Ce6 hybrid hydrogel was successfully used to improve PTT/PDT through ameliorate tumor hypoxia environment [53]. After, as-synthesized hybrid hydrogel was injected into the tumor areas, exhibiting excellent biocompatibility and biodegradability, especially when it was precisely introduced into the innermost. Further, MnO_2_ and Ce6 can be continuously permeated into the surrounding environment by softening and hydrolyzing hybrid hydrogel. More importantly, SH as light absorber converts light into thermal under laser irradiation, thus hydrogel itself can be applied in PTT. What’s more, MnO_2_ released from hydrogel can catalyze excessive H_2_O_2_ in tumor tissues to generate oxygen, which can enhance PDT outcomes upon being exposed under 660 nm laser and attenuate tumor hypoxia environment. This multifunction agarose@SH/MnO_2_/Ce6 hybrid hydrogel was injected into the tumor sites without entering the circulatory system, which help avoid potential biohazard and being cleared by the body immune system. Therefore, it achieves “one injection, multiple therapies”, and inspires us to exploit suitable hydrogel-based approaches to various disease therapy in clinic.

### Perspectives

Thermal therapy has the advantages of minimal invasiveness and high selectivity, which is an effective strategy for tumor therapy in clinic [54, 55]. Compared with conventional approaches, thermal therapy can precisely control local temperature and effectively avoid unnecessary side effects such as damaging normal issues and destroying body immune system [56]. When the cell temperature reaches 41 °C, cell becomes temporarily inactive and causes protein denaturation, and this condition lasts for several hours. As the temperature arrives at 43 °C, it may cause long-term cells inactivation. Although thermal therapy has achieved much exciting progress in the field of tumor therapy, there is still a lack of safe and effective photothermal agents or drug carriers with good biocompatibility and biodegradability.

Hydrogel is the ideal candidate for drugs carrier with good biocompatibility and biodegradability in current tumor treatment. Incorporating inorganic/organic into hydrogel has attracted widely attention due to their cooperative effects which can enhance therapy effects against tumor. Among various responsive hydrogel, thermosensitive hydrogel can precisely and continuously control drug release through temperature stimulus in tumor tissues. Compared with percutaneous and intravenous injection methods, the accurate locate injected administration hydrogel within the agents has better biosafety in vivo [57].

## Conclusions

Despite the significant merits of hydrogel, the clinical application has been limited due to unsatisfactory biodistribution, poor biocompatibility, and poor tumor penetration ability. In this article, thermosensitive hydrogel has the advantages of better biocompatibility, excellent tumor inhibit ability, and no unnecessary side effects. These merits will further promote their application in clinic for various disease treatment.

## Data Availability

Not applicable.

## Abbreviations

DT-PEG: Difunctional telechelic poly (ethylene glycol)
FVIOs: Ferromagnetic vortex-domain iron oxide (FVIOs)
PTT: Photothermal therapy
Pdots: Polymer dots
NIR: Near-infrared
PAI: Photoacoustic imaging
CT: Computed tomography
PDA: Polydopamine
N-HA: Nano-hydroxyapatite (n-HA)
DDP: Immobilize cisplatin
CS: Chitosan (CS)
OSA: Oxidized sodium alginate
DDP: Cisplatin
HA: Humic acid
HA: Humic acid
PDT: Photodynamic therapy
ROS: Oxygen reactive species
PS: Photosensitizers
LMP: Low melting point
SH: Sodium humate
Ce6: Chlorin e6
MnO_2_: Manganese oxide
